# An adjunction hypothesis between qualia and reports

**DOI:** 10.3389/fpsyg.2022.1053977

**Published:** 2023-01-30

**Authors:** Naotsugu Tsuchiya, Hayato Saigo, Steven Phillips

**Affiliations:** ^1^School of Psychological Sciences and Turner Institute for Brain and Mental Health, Monash University, Melbourne, VIC, Australia; ^2^Center for Information and Neural Networks (CiNet), National Institute of Information and Communications Technology (NICT), Suita, Japan; ^3^Advanced Telecommunications Research Computational Neuroscience Laboratories, Kyoto, Japan; ^4^Nagahama Institute of Bio-Science and Technology, Nagahama, Japan; ^5^National Institute of Advanced Industrial Science and Technology, Tsukuba, Japan

**Keywords:** qualia, consciousness, report, adjunction, category theory

## Abstract

What are the nature of the relationship among qualia, contents of consciousness, and behavioral reports? Traditionally, this type of question has been only addressed *via* a qualitative and philosophical approach. Some theorists emphasize an incomplete and inaccurate nature of reports of one's own qualia to discourage formal research programs on qualia. Other empirical researchers, however, have made substantial progress in understanding the structure of qualia from such limited reports. What is the precise relationship between the two? To answer this question, we introduce the concept of “adjoint” or “adjunction” from the category theory in mathematics. We claim that the adjunction captures some aspects of the nuanced relationships between qualia and reports. The concept of adjunction allows us to clarify the conceptual issues with a precise mathematical formulation. In particular, adjunction establishes coherence between two categories that cannot be considered equivalent, yet has an important relationship. This rises in empirical experimental situations between qualia and reports. More importantly, an idea of adjunction naturally leads to various proposals of new empirical experiments to test the predictions about the nature of their relationship as well as other issues in consciousness research.

## 1. Introduction

What is the nature of the relationship among consciousness, brain, and behavior? What can science reveal about these relationships? Philosophers have raised these important questions and have been debating on possible answers based on logic and arguments. Some researchers have concluded pessimism toward research on subjective consciousness, arguing that more productive research can arise by focusing on brains and behaviors. This line of thought is intensified by behaviorists, leading to the decline of studies of consciousness in the middle of the twentieth century (Baars, 2009).

With the cognitive revolution and the invention of the brain imaging, the relationship between the brain and consciousness has become the active target of research over the last 30 years. The empirical research program in trying to find the neural correlates of consciousness, the NCC, has made substantial progress to the point that we now need a theory to coherently understand these findings (Del Pin et al., 2021; Doerig et al., 2021; Seth and Bayne, 2022).

Some of these theories try to deal with the relationship between neural activities and perceptual consciousness. Among them, the most successful and influential has been the global neuronal workspace theory (Dehaene and Naccache, 2001; Mashour et al., 2020). In most cases, however, these theories have tried to explain the results of the empirical studies that tried to capture perceptual consciousness in a binary response: seen vs. not seen or seeing A or B. This is partly due to the fact that many neuroscientific studies have tried to find the neural correlates of consciousness [the NCC (Koch et al., 2016)] using the experimental situation where conscious experience fluctuates from one to the other under the condition of constant sensory input. This strategy has allowed the researchers to dissociate the neural activity that merely correlates with sensory inputs from those that are more closely correlated with the contents of consciousness.

While this strategy has been productive, there remains a core enigma in understanding the relationship between the brain and consciousness. Our conscious experience is not binary. It consists of various modalities (e.g., vision, audition, and touch) that compose a unified experience at any moment (Balduzzi and Tononi, 2009). Each modality, such as vision, consists of submodalities, color, shape, and motion, in space. How does the quality of color, for example, as uniquely experienced and so different from other experiences (e.g., motion or audition) arise from a seemingly uniform neural mechanism? The questions, related to the quality of consciousness, are sometimes called the problem of “qualia” (its singular “quale”) (Kanai and Tsuchiya, 2012). Throughout this article, we use the term “qualia” to refer to the quality of contents of consciousness. Qualia seem impossible to deal with the binary response paradigms in the NCC framework.

Recently, there have been some proposals to approach the relationship between qualia and the brain from a structural viewpoint (Haun and Tononi, 2019; Fink et al., 2021; Lee, 2021). As a structural approach to qualia, we ourselves also promoted the use of category theory (Tsuchiya et al., 2016, 2022; Tsuchiya and Saigo, 2021). Category theory is a mathematical tool specifically invented to deal with structure (Mac Lane, 1998). Category theory allows us to characterize a particular quale as a collection of relationships with other qualia *via* the Yoneda lemma (Tsuchiya et al., 2016) even with a graded level of relationships, such as perceptual similarity, using enriched categories (Tsuchiya et al., 2022). For other category theoretical approaches to consciousness, refer to Kleiner and Tull (2021), Northoff et al. (2019), and Signorelli et al. (2021).

In this article, we will deal with the problem of incomplete and inaccurate reports about qualia by introducing a concept in category theory, called “adjunction”^1^. Intuitively, adjunction is a structural relationship between two categories that are coherently related but does not correspond to each other in a one-to-one way. An example is a relationship between a category of real numbers (*R*) with “no larger than” relationships (≤) and a category of integers (*Z*, ≤). They are obviously not the same, but there is a significant and coherent relationship, called adjunction. Adjunction is an important concept that was formally defined in category theory. According to Mac Lane, who is a founder of category theory, “adjoints are everywhere (p. 97) (Mac Lane, 1998)”. Yet, as far as we know, this “everywhere” has not included consciousness research yet. The expansion of the application of adjunction into consciousness research is one of the goals of this article. For an application of adjunction to the dual process theory of cognition, refer to Phillips (2018).

With the concept of adjunction, some controversial topics in consciousness research may be better conceptually analyzed. Some exemplar debates include the following: debates on the richness of a moment of conscious experience (Block, 2007; Kouider et al., 2010; Lau and Rosenthal, 2011; Vandenbroucke et al., 2014; Cohen et al., 2016; Haun et al., 2017; Bronfman et al., 2019), possibilities of correspondence in color qualia between people (Palmer, 1999), and private nature of reports on qualia (Dennett, 1988; Piccinini, 2009). We hope that our adjunction hypothesis clarifies the conceptual confusion in these continuing debates.

We do not claim that a concept of adjunction solves these debates. Rather, our idea can lead to various empirical research programs as described in the Discussion. Concepts of adjunction also help us to understand why, despite some theoretical worries, empirical research has made substantial progress in understanding the structure of qualia from such limited reports as we elaborate this in the Discussion.

To benefit from conceptual clarification and ideas for future research, it is crucial to understand the precise mathematical formulation of adjunction. In the next section, we quickly review basic concepts in category theory. We use the examples of color qualia with relationships of similarity (~) and intensity (≤) to explain the concepts of categories, functors, natural transformations, the equivalence of categories, and, finally, adjunction. Then, we consider some empirical experiments based on the concept of adjunction.

## 2. Category theory concepts

In simple terms, two systems may be related to an adjoint manner even when they are not strictly the same, so that when one cannot find the exact correspondence between the two. Such a situation arises frequently in mathematics [e.g., consider a category of integers (Z, ≤) and a category of real numbers (R, ≤)]. However, for two systems to be linked by an adjunction, the deviations in the maps between them must be related in a systematic and coherent way. In an example of integers and reals, through the relationship of “ceiling,” one can always approximate any given real number with the smallest integer that is bigger or equal to the real number.^2^

Our hypothesis is that a category of qualia is related to a category of reports in adjunction. They are not the same, but their relationship is coherently explained. This fits with our intuition that each of us can report some aspects of our qualia quite confidently with a reasonable degree of accuracy. For example, when we see a patch, such as ■, we never report “I saw something white.” This is also consistent with the general consensus in consciousness research that what participants report should be taken seriously unless there is strong evidence to doubt their remarks [e.g., Anton's blindness (Sackeim et al., 1979), also refer to Koch and Tsuchiya (2008)]. This nuanced, yet coherent, relationship between qualia and reports can be precisely understood as an adjunction. In Discussion, we argue that this formal understanding of the adjunction between qualia and reports would lead to empirical experimental paradigms to be tested in the future.

To introduce adjunction, we need several category theory concepts. We present them with examples that are relevant to consciousness research. We also recommend the following textbooks as an introduction to category theory: formal (Mac Lane, 1998; Leinster, 2014), conceptual (Lawvere and Schanuel, 2009; Simmons, 2011), philosophical (Krömer, 2007; Marquis, 2008), applied (Spivak, 2014; Fong and Spivak, 2019), and computational (Walters, 1991; Bird and De Moor, 1996). We have also provided introductory tutorials online (Phillips, 2022a; Tsuchiya, 2022). For the latest introduction of category theory to cognitive scientists, refer to Phillips (2022b).

Finally, we recommend beginners of category theory to draw figures in this article by themselves. Drawing each component of figures (e.g., an arrow) is one of the most effective ways to understand the category of theoretical concepts.

### 2.1. Category, using an example of color qualia

#### 2.1.1. Definition: Category

A category is a collection of objects and arrows that satisfies the following axioms.

1) An arrow, f, has a source and a target object (f:A → B).
2) If two arrows, f and g, share an object B as a target and a source, f and g can be composed to form another arrow f;g. (f:A → B, g:B → C, then f;g: A → C).
3) If three arrows f, g, and h are composable, the order of composition does not matter, that is, (f;g);h = f;(g;h).
4) Each object A is associated with an identity arrow labeled 1A whose role for composition is analogous to the role of the number 1 for multiplication. Compare 1A; f = f = f;1B with 1 x X = X = X x 1, hence the notation 1A for an identity arrow.

The following examples are explained in more detail in our previous article (Tsuchiya and Saigo, 2021).

#### 2.1.2. Example: Category (Q, ~)

Consider a collection of color qualia, Q, and their relationship as “indistinguishably similar: ~.” Then, this constitutes a category of qualia (Q, ~) and satisfies all the above conditions.

#### 2.1.3. Example: Category (Q, ≤)

Now, consider the same objects as (Q, ~) but with a relationship as “not darker than: ≤.” This constitutes another category (Q, ≤). The difference between (Q, ~) and (Q, ≤) will become important when we introduce the equivalence of categories and adjunctions later.

#### 2.1.4. Example: Behavioral reports (B, ~), (B, ≤)

Consider a range of behavioral response options, B. B's objects can be various button press options or linguistic response labels, such as {“red,” “blue,” “green,” “black,” “gray,” and “white”}. As each label is distinguishable, for (B,~), there are no “~” relationships among any objects. Yet, each object has a self-referential arrow to itself. For (B, ≤), at least, we can order “white” ≤“gray” ≤“black”.^3^ In both cases, we consider a situation where a relationship (~ or ≤) either exists or does not. A category where two objects have at most one arrow is called “preorder.” We rely on the preorders when we explain the following concepts, such as functors, natural transformations, and adjunctions, as they simplify the explanations.

### 2.2. Functor: “Respond” and “Infer” between categories of qualia and behavioral reports

Two categories are related by maps, called functors, that send the objects and arrows in one category to the objects and arrows in another category in a way that is structurally consistent (refer to definition). As stated later, adjunctions relate two categories that satisfy conditions that are stronger than functoriality. An adjunction requires two categories to be related *via* two functors, where one functor maps a category to the other category, while the other functor is in a reverse direction with some coherence condition as we explain later.

#### 2.2.1. Definition: Functors

A functor F maps the objects and arrows in category C to another category D in a coherent manner. Functor F satisfies the following two conditions:

1) F(f);F(g) = F(f;g), that is, it preserves composition; and
2) F(1X)=1F(X), that is, it preserves identity.

A functor from/to the same category C (i.e., F: C → C) is called an endofunctor (see below).

#### 2.2.2. Example: A functor “Respond,” which maps experienced qualia (Q, ~) into behavioral reports (B, ~)

Consider a collection of reddish patches, such as ■ and ■, where all objects are “indistinguishably similar, ~.” In such a category (Q, ~), all objects are connected through arrows (~). A functor “Respond” maps all objects in Q to one object “red” in B and all arrows ~ in Q to the identity ~ in B. This satisfies the above conditions. Similarly, a functor “Respond” can map the achromatic color qualia category with the “not darker than” arrow, (Q, ≤), into another category, (B, ≤) with the level of darkness, such as {“black,” “gray,” and “white”}.

#### 2.2.3. Example: A functor “Infer,” which maps behavioral reports (B, ~) or (B, ≤) into representative qualia

Now, we consider a functor, which maps category B into Q. An intuitive and simplest functor is the one that picks up the most representative color qualia based on the response labels. For example, a functor “Infer” maps (B, ≤) into (Q, ≤), where a label “black” is mapped to ■ and so on. Note that this also preserves the relationships “ ≤” in B into “ ≤” in Q.

#### 2.2.4. Example: An endofunctor and the identity functor 1F

A functor from a category C to the same category C (i.e., a functor of form F: C → C) is called an endofunctor. An important special case of an endofunctor, called the identity functor, maps each object to itself and each arrow to itself. As we use 1X to denote the identity arrow for an object X in category C, we denote the identity functor from category C to category C as 1C. We will use these concepts later to define the equivalence of categories and clarify their difference from an adjunction.

### 2.3. Natural transformation

Natural transformation is a key concept in category theory, which is necessary to precisely define various degrees of sameness between different categories.

#### 2.3.1. Definition: Natural transformation

**Figure 1:**
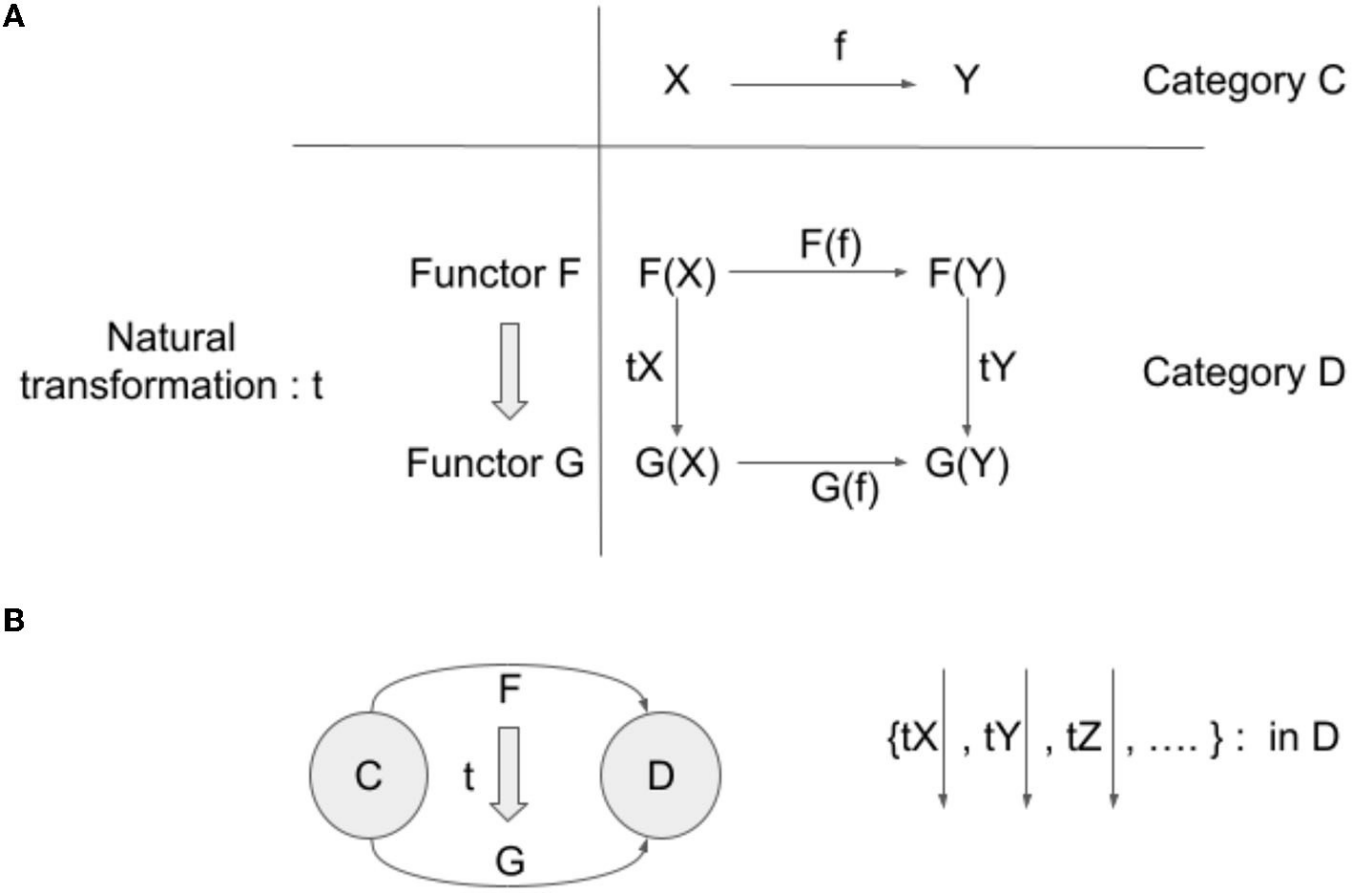
**(A)** Natural transformation and its coherence condition. **(B)** Natural transformation can be considered as an arrow between two functors, which have the same domain and codomain. Natural transformation is a collection of arrows in the target category of the underlying functors.

Suppose two functors from a category C to a category D (F: C → D, G: C → D). A natural transformation t is a family of arrows in D {tX: F(X) → G(X), where X is an object in C}. That is, for each object X in C, there is an assigned arrow tX: F(X) → G(X) in D, such that for each arrow f: X → Y in C, the following equality is satisfied: F(f);tY = tX;G(f) (refer to Figure 1).

#### 2.3.2. Example: Functors “Coarse” and “Fine” from category (Q, ≤) to category (B, ≤)

Suppose the preordered set of achromatic qualia (Q, ≤) and the preordered set of behavioral responses (B, ≤) as categories. The objects of Q are {■, ■, and so on} and the objects of B are “black,” “blackish gray,” “whitish gray,” and “white.” Suppose the two functors from Q to B, the functor “Coarse” maps each object in Q to either the object “black” or the object “white” in B. The functor “Fine” maps each object in Q to an object in B.

**Figure 2:**
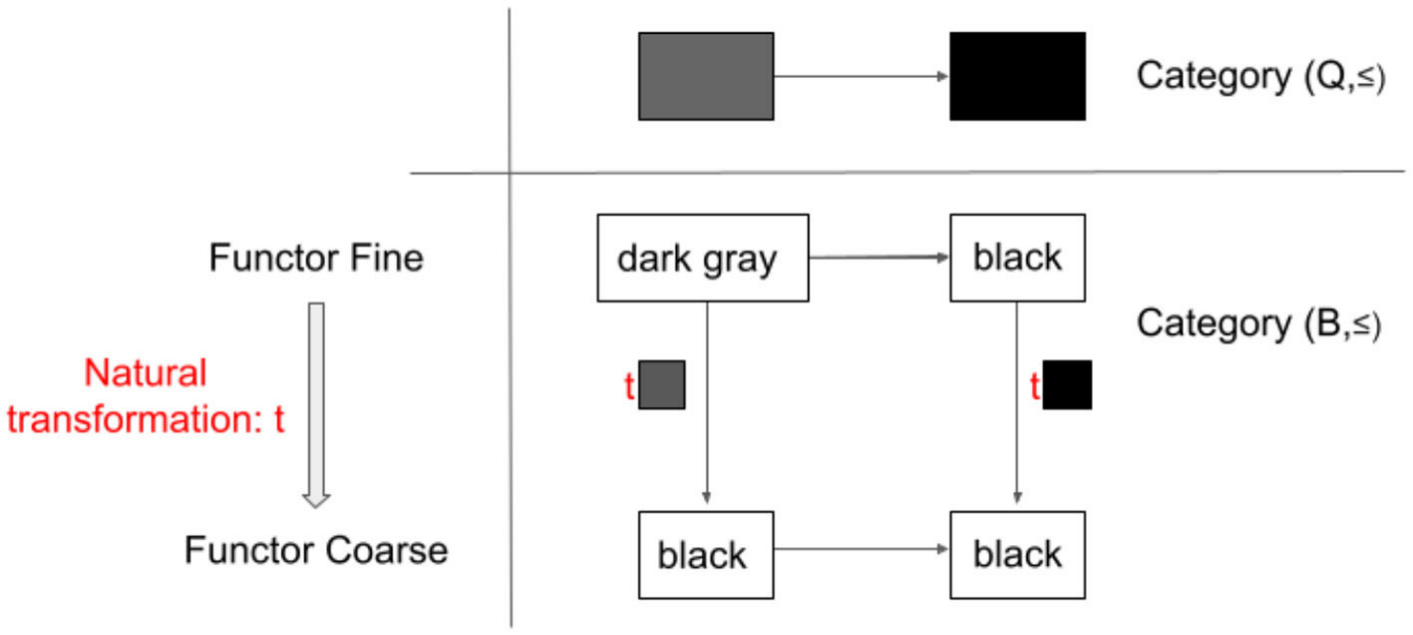
The natural transformation between two functors, “Fine” and “Coarse,” each of which maps category (Q, ≤) into category (B, ≤).

Figure 2 shows the existence of a natural transformation, t, from a functor “Fine” to “Coarse.” What does this mean? The naturality condition for t is depicted in the bottom panel of Figure 2.

First, let us confirm how functors fine and coarse map category Q to B. Fine maps ■ to “dark gray” and ■ to “black.” The arrow between them in (Q, ≤) is preserved in (B, ≤). Similarly, Coarse maps both ■ and ■ to “black” and their arrow to ≤.

Second, let us check whether each object in Category Q has a corresponding arrow in Category B. Note that this is a correspondence between the objects to arrows. If it exists, each of these arrows is a component of natural transformation. In this case, as Category B's arrow is only ≤, it means that each object in Q has an arrow ≤, such as t■, t■, and so on. It turns out that each mapped object by Fine has an arrow ≤to the corresponding mapped object by Coarse.

This in turn means that the condition for natural transformation is satisfied. From “dark gray” on the top left, there are two pathways to “black” on the bottom right. Through the top right, we go through “dark gray” ≤“black” ≤“black”. The second ≤is a ■ component of natural transformation t,t■. Through the bottom left, we go through “dark gray” ≤“black” ≤“black,” where the first ≤is a ■ component of natural transformation t,t■.

It is important to note that there is no natural transformation from Coarse to Fine. If you swap the top and the bottom row of Figure 2, you will see that no arrow can go from “black” to “dark gray”, and thus, there is no t■ component of natural transformation.

#### 2.3.3. Example: Functors Coarse and Fine from category (Q, ~) to category (B, ~)

It is instructive to consider what happens when we switch arrows in both Q and B with the “indistinguishable, ~” relations. Here, all arrows are invertible. If two qualia ■ and ■ are indistinguishable, then they have an arrow in between. Then, a functor Coarse should map them into the same behavioral label “black.” Likewise, a functor Fine should also map both to “black.” Then, there exist natural transformations between the functors Coarse and Fine. In this case, the natural transformation itself is invertible. Such an invertible natural transformation is called natural isomorphism or natural equivalence.

### 2.4. Graded level of sameness from identity, isomorphism, equivalence, and adjunction

The above explanation motivates our need to introduce the definition of adjunction. To appreciate the value of the weaker but more versatile form of sameness, adjunction, we formally introduce stronger notions of sameness in category theory; identity, isomorphism, and equivalence. These strong notions of sameness have been invoked in the context of consciousness research (Palmer, 1999; Oizumi et al., 2014; Myin and Zahnoun, 2018; Fink et al., 2021). While adjunction is substantially weaker than these strong notions of sameness, it is much more strict than the mere presence of functors (in both directions). Every identity functor is an isomorphic functor, but an isomorphic functor need not be an identity functor. An adjunction is (systematically) weaker in the sense that the isomorphisms are between qualia and report relations (called their hom-sets), but not necessarily the individual qualia and reports. We suspect that adjunction is likely to be the more appropriate level of sameness, both conceptually and empirically in the context of consciousness research.

“Identity” is the strongest and it does not apply to the case of Q and B obviously. When we already “distinguish” categories of qualia and reports, they are not identical.^4^

### 2.5. Isomorphism of categories

“Isomorphism of categories” is still very strong. Two categories are isomorphic when there exists a functor that has its inverse.

#### 2.5.1. Definition: Isomorphism of categories

Categories C and D are isomorphic when there exists a pair of functors F and G; F:C → D and G:D → C, where F;G = 1C and G;F = 1D. 1C and 1D are the identity functor (Section Functor: “Respond” and “Infer” between categories of qualia and behavioral reports), which maps every object and arrow in itself.

#### 2.5.2. Example: Category of primary color qualia (PQ, ~) and behavioral responses (B, ~) and functors respond and infer

Let us consider a category of qualia, consisting of qualia of “primary” colors, such as ■■■ with “indistinguishably similar” as a relationship, which we denote as (PQ, ~). As another category, we consider a category of behavioral reports, which consist of basic color terms, such as “red,” “blue,” and “green” with indistinguishability, ~, as arrows. The functor Respond maps each primary color quale to the corresponding basic color term. The functor Infer maps each term into a primary color quale. When we compose these functors, Respond;Infer, this results in the identity functor, 1PQ, which maps category PQ to PQ. We can easily confirm the other direction, that is, Infer;Respond = 1B.

This simple example makes it clear that the “isomorphism of categories” is very strict. Isomorphism requires that each object (or arrow) in either category needs to be mapped back into the original object (or arrow) through mapping into the other category. If there is any loss of information (e.g., coarsening or categorization), this does not work.

Such loss is inevitable when one considers the relationship between qualia and behavioral reports in almost any case. To achieve isomorphism, we had to remove gradation in color qualia, such as ■. If there is gradation, which collapses several qualia into one behavioral response label, then, isomorphism does not work. If we start from a graded quale, ■, which is mapped to “red” by a functor Respond, then, a composite functor Respond;Infer will map it back to a primary color such as ■.

### 2.6. Equivalence of categories

To resolve this, we introduce a weaker concept of sameness; “equivalence of categories.” The equivalence of categories is an essential sameness between two categories, which may not be intuitively similar at the surface level. Depending on the type of arrows, categories of qualia and behavioral response can be equivalent. Equivalence is defined by employing a notion of natural transformation (Section Natural transformation).

#### 2.6.1. Definition: Equivalence of categories

Categories C and D, which are related to by two functors F (F:C → D) and G (G:D → C), are equivalent if there exist two invertible natural transformations t: 1C → F;G and s: G;F → 1D. Invertible natural transformations are called natural isomorphisms.

#### 2.6.2. Example: Categories of color qualia (Q, ~) and behavioral responses (B, ~) and functors Respond and Infer

By introducing the equivalence of categories, we can include a range of color qualia, not limited to primary colors, unlike the case for isomorphism of categories. Consider a color qualia category allowing gradation. Functor Respond collapses a shade of red, such as ■ and ■, into one response label “red.” Functor Infer will map a basic color term, such as “red,” into the corresponding focal color, such as ■. Now, we examine whether a composite of functors Respond;Infer and Infer;Respond is naturally isomorphic to 1Q and 1B, respectively.

The latter case is easy to see. Starting from any color label object in B, we can pick the primary color quale and come back to the original color label object. All arrows in (B, ~), are conserved. Thus, a functor Infer;Respond is indeed the identity functor, 1B. The arrow between Infer;Respond and 1B is an invertible natural transformation or natural isomorphism.

**Figure 3:**
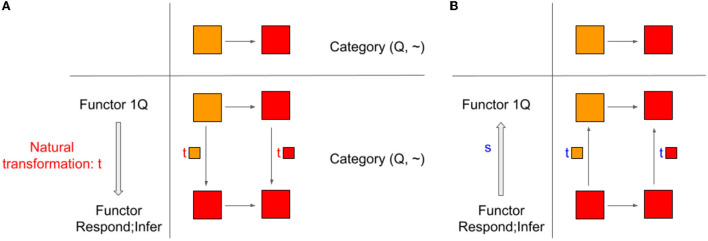
**(A)** Natural transformation t from the identity functor 1Q to a functor Respond;Infer is a natural isomorphism when the underlying category is (Q, ~). But it is not if the underlying category is (Q, ≤). **(B)** Natural transformation s from the functor Respond;Infer to the identity functor 1Q.

The former case is also easy but requires some thought. Starting from ■ in Q, Respond will map it into “red,” and then, Infer will map “red” back to ■. Thus, the functor Respond;Infer loses some information. But that is fine. The important “information” that “equivalence” tries to keep is the “relational structure” of Q, that is, embodied by the arrows, ~. As seen in Figure 3 below, in this case, natural transformation works in an invertible way. This is precisely because there exists an arrow from ■ to ■ (t■ component of natural transformation t:1Q → Respond;Infer in Figure 3A) as well as an arrow from ■ to ■ (s■ component of natural transformation s: Respond;Infer → 1Q in Figure 3B).

What happens if we replace (Q, ~) with (Q, ≤)? That would keep all the objects the same, but we now consider a structure that is characterized by “not darker” relations. Critically, now, not all arrows in Category Q are invertible. Thus, a functor Respond;Infer has no natural transformation to the identity functor 1Q if the underlying category is (Q, ≤). How can we characterize a relationship between qualia and reports in this case? The answer is adjunction.

### 2.7. Adjunction

We introduce a definition of adjunction using a concept called “universal morphisms”.^5^ This definition makes it easy to see the relationship between the adjunction and equivalence of categories. Though this definition may look different from textbook definitions of adjunction, mathematically speaking, they are all equivalent.^6^

#### 2.7.1. Definition: Adjunction

An adjunction consists of a pair of functors F: C → D and G: D → C and a natural transformation t: 1C → F;G such that for each object X in C, the pair [F(X), tX: X → F;G(X)] is a universal morphism from X to G, i.e., for every object Y in D and morphism f:X → G(Y) in C, there exists a unique morphism g: F(X) → Y in D such that f = tX ; G(g).

Let us decompose this definition to see a clear connection to the equivalence of categories. Consider Categories C and D, which are related by two functors F (F:C → D) and G (G:D → C). There exists a natural transformation, t: 1C → F;G. Up to here, the definition is similar to the equivalence of categories, but this t does not have to be a natural “isomorphism.” That is, t does not have to be “invertible.” Note that for this definition, we also do not need the other side of natural “isomorphism” s: F;G → 1D. What is the consequence of this difference?

Let us come back to the latter part of the definition. It means that if you pick any X and any arrow f: X → G(Y) in Category C, there is a unique arrow g:F(X) → Y in Category D, such that f=tX;G(g). This part appears most complicated among the concepts encountered in this article. One novel concept in the second part is the “uniqueness” of the arrow. We simplify this by considering the “preorder” category, where there is only one type of arrow.

In sum, like the case of equivalence of categories, when we start with a particular object in one category, mapped to the other *via* a functor, then back to the original category *via* another functor, we may not arrive at the original object.

**Figure 4:**
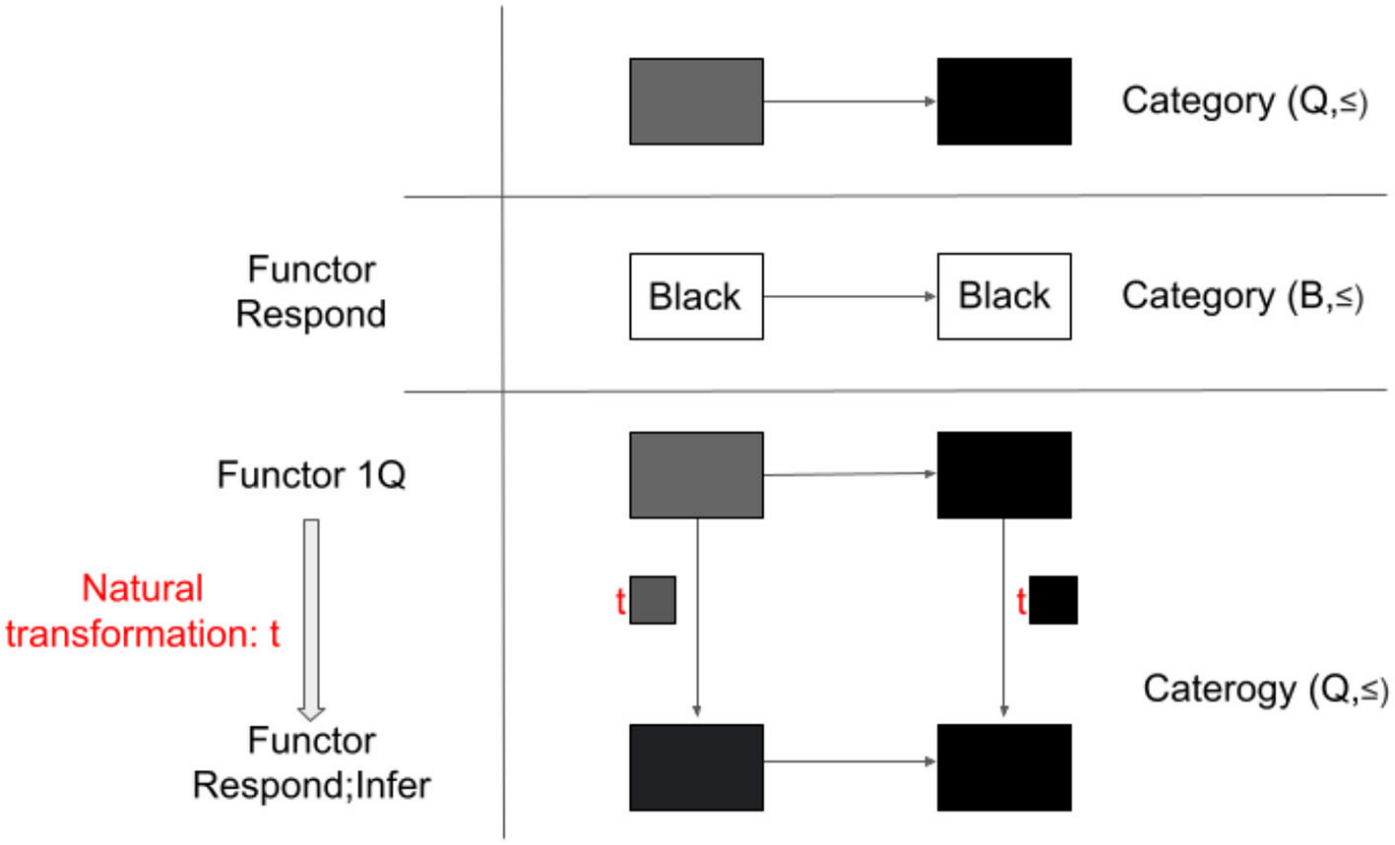
Adjunction. Category (Q, ≤) and Category (B, ≤) are related in a sense of adjunction with functors Respond and Infer, though not categorically equivalent.

With this, let us go through the example in Figure 4 below to understand the definition.

#### 2.7.2. Example: Categories of color qualia (Q, ≤) and behavioral responses (B, ≤) and functors Respond and Infer

We quickly confirm that the first part of the definition for adjunction is satisfied. Category (Q, ≤) and Category (B, ≤) are related by Functor Respond, which maps both ■ and ■ to the behavioral response label “black.” The arrow ≤is preserved. Natural transformation s:Infer;Respond → 1B is easy to check (the same procedure as in the example for equivalence). Natural transformation t: 1Q → Respond;Infer also exists. Note that this natural transformation is not invertible, which makes Category (Q, ≤) and Category (B, ≤) not categorically equivalent, as we confirmed in the last example.

Now, the second part. Consider an object ■ and an arrow ≤_Q_: ■ → ■ (which means that ■ is not darker than ■). For the sake of clarity, here, we made it explicit that the arrow, ≤_Q_, is in Category Q. Here, we chose ■ as G(Y) to satisfy this arrow condition. As long as this arrow condition is satisfied, any qualia can be chosen as G(Y) in Q.

Then, the requirement of adjunction says that there exists a unique arrow in B, which satisfies a certain condition. Now, we are considering a category with only one arrow, ≤_B_, (preorder), any arrow in B is unique. This arrow needs to satisfy ≤_B_: Respond(■) → Y. Respond(■) is “black.” So, Y is the behavioral label that satisfies ≤_B_: “black” → Y. Obviously, Y can be “black.”

Finally, this arrow in B, ≤_B_: Respond (■) → “black” should now satisfy the condition in the original qualia category, such that ≤_Q_ = t■; Infer( ≤_B_). What does this mean?

The arrow on the left-hand side, ≤_Q_, is the original arrow that we considered in Q (i.e., ≤_Q_: ■ → ■), which is the same as the composite of the top left → top right → bottom right pathway of the natural transformation in Figure 4.

The arrow on the right-hand side t■;Infer( ≤_B_) is a composite of t■ and Infer( ≤_B_). As can be confirmed in Figure 4, t■ is just an arrow from ■ [=1Q(■)] to ■ [=Respond;Infer(■)]. Recall that Infer maps ≤_B_ to ≤_Q_. Thus, the above corresponds to the top left → bottom left → bottom right pathway of the natural transformation in Figure 4. Thus, ≤_Q_ = t■; Infer( ≤_B_) is satisfied.

In this case, there is a natural transformation from the identity functor 1Q to a composite functor Respond;Infer. (Same goes with a composite Infer;Respond to the identity functor 1B, not shown in Figure 4). Critically, for any object X (e.g., ■) and any arrow f:X → Infer(Y) in Q [e.g.,■ ≤Infer(black)], there is a unique arrow g:Respond(■) → Y, in this case, Respond(■)=black ≤black=Respond(■), such that f =tX;G(g).

Taken together, Categories (Q, ~) and (B,~) are equivalent while Categories (Q, ≤) and (B, ≤) are not. The latter is still formally related through adjunction.

## 3. Discussion

In this section, we revisit some of the conceptual issues in consciousness research that can be clarified by the concept of adjunction. Along the way, we will propose some possible research programs with empirical experiments.

### 3.1. Conceptual clarification

Historically, various philosophers and scientists have pointed out the problem of incompleteness and inaccuracy in introspective reports. That is, when we are presented with a stimulus, reporting on it appears to contain some levels of ambiguity. One issue with this observation is whether our conscious experience should directly and veridically reflect what is presented. This is an issue of a philosophical option of “direct realism” (Genone, 2014). As the focus of our article is not to discuss various philosophical options, we do not go into the details of the debate in this direction (for related views, refer to Prakash et al., 2020).

For example, Wundt and Fechner's psychophysics program assumed that some aspects of mental phenomena (e.g., visual perception) should be highly veridical while others (such as emotional experience) may not be (Käufer and Chemero, 2021). In this program, researchers identified various factors that degrade initially perfect sensory representations such as noise, distraction of attention and loss of memory. Rigorous psychophysics arrived at a concept of just noticeable differences as perceptual “atoms,” trying to explain the entire experience building on this concept. While successful in some simple perceptual domains, this type of approach is limited in application. The approach is especially difficult to apply to more complex structures, such as conscious experience as a whole, which may consist of experiencing various objects at the same time across different spatial locations and so on. Furthermore, perception is known to interact with internal mental processes, such as attention, memory, and emotion. Thus, if we are to characterize the structure of our conscious experience or qualia, we would need an alternative approach.

What we proposed in this article is a philosophically neutral and general framework. With a concept of equivalence and adjunction, some types of qualia can be coherently related to what is introspectively examined and behaviorally reported. Under some experimental contexts, some simple qualia may be precisely reported with the appropriate behavioral labels. This can be formulated as an original qualia object X in category Q, which is transferred to a behavioral report object in B *via* Functor Report, then transferred back to another object X' in category Q *via* Functor Infer. Under some situations (e.g., rigorous psychophysics with small response alternatives), any difference between X and X' can be made very small. Under more naturalistic situations (e.g., living in a complex world with attention shifting and lapsing and memory decaying), X and X' can differ substantially. Even with the latter case, however, X and X', we argue, are coherently related.

In science, a dominant mode of progress is to pursue minimization of the deviation between X and X' through technological development (e.g., better equipment, analysis). However, other approaches are also possible and, in fact, can be more important, especially, in the fields of psychology and cognitive science. Studies of attention and memory can be seen as an attempt to characterize how objects and arrows in Q are systematically affected in B. For example, Functor PayAttentionCenter may preserve the objects and arrows in Q at fovea at the expense of those at the periphery, while PayAttentionPeriphery does the opposite (Carrasco, 2011). As the studies of a functor Infer, we can consider various effects of “cognitive bias” (Kahneman, 2011). Under certain situations, we infer (or imagine) what the prototype of the label is with a systematic bias. This bias in inference may generate large deviations from X to X'. In other words, various sources of deviations can be analyzed in terms of functors as well as the structure of categories.

Our adjoint functor approach provides this type of conceptual clarity to consciousness research. Even if qualia and behavioral reports are not identical, isomorphic, or equivalent, adjunctions may provide a coherent, valid, and appropriate structural description between them. In Sections Equivalence of categories and Adjunction, we considered two categories (Q, ~) and (Q, ≤). They consist of the same objects with different arrows. We found the former (Q, ~) to be categorically equivalent to (B,~). This means that when qualia and behaviors are considered with a relationship of “indistinguishably similar, ~,” graded qualia can be collapsed into some discrete behavioral labels in B, without losing the essential structural properties in Q. This can apply to a large class of qualia where similarity matters for its characterization. Meanwhile, we found that (Q, ≤) is not equivalent to (B, ≤), but they participate in an adjunction. This is important, as an ordered relationship ≤is fundamental to almost all the aspects of qualia. The intensity of various qualia,^7^ inclusion relationships among spaces (Koenderink et al., 2014; Haun and Tononi, 2019; Prentner, 2019), and so on have been discussed intensely.

Generally speaking, ordered relationships together with chunking of elements would constitute adjoint relations. This involves a large class of cognitive operations to construct an equivalence class as in categorization. Integer and real numbers are easy examples in mathematics. More fundamental mathematical relationships in adjoint include the following: categories of Sets and Categories, Free and Forgetful construction, and universal morphisms, limits, and colimits (Mac Lane, 1998; Lawvere and Schanuel, 2009; Simmons, 2011; Leinster, 2014). More informally, the concept of adjunction also applies to the relationships between special vs. general, known vs. unknown, and so on (Ojima, 2005). So far, these concepts have been employed to explain cognitive functions, rather than experiential aspects. We believe that these are powerful conceptual tools, which can offer more flexibility and rigor in a qualitative and quantitative manner, for future consciousness research (Krömer, 2007; Marquis, 2008; Landry, 2017).

### 3.2. Qualia reconstruction in the Sperling paradigm: Proposals for empirical experiments with broad-sense qualia

So far, we mostly focused on our discussion on qualia in a narrow sense (Balduzzi and Tononi, 2009; Kanai and Tsuchiya, 2012). Narrow-sense qualia refer to a particular aspect of a moment of experience, such as the redness of the apple. Broad-sense qualia are the entire experience at a given moment. We believe that a concept of adjunction can deal with both senses of qualia and their relationship with behavioral reports. Consider a moment of experience, which is sometimes considered a quale in a broad sense. Let X be a moving experience that you might have in front of the ocean at the sunset beach. X can be reported in various ways. Based on the reports, we can infer what it was like as X'. That is, Report;Infer(X)=X'. In this case, X' cannot be an experience that you may have in a dense and dark jungle at night. There can be much more reasonable Report and Infer functors that assure the adjunction between qualia and report.

As one future research program, we consider promising a qualia reconstruction paradigm, which explicitly measures the deviation from the original object X to the reconstructed object X'=Report;Infer(X). To be more concrete, we briefly introduce one of the most controversial experimental paradigms in consciousness research: the Sperling paradigm (Sperling, 1960).

In the Sperling paradigm, one's subjective and vivid impression of “I saw everything” is betrayed by their objective performance of poor reports of a few. What we can access and report appears highly limited and less certain. However, when participants are asked to report some specific letters' location and identity AFTER the letters disappear from the display, their performance improves substantially, to the extent that behavioral performance accurately reflects the initial impression of “I saw everything.” This seems to suggest that, somehow, the initial broad-sense quale can lead to an accurate report. While accessing the detailed information, however, initial information may be lost.

This is just one of many interpretations of the results of the Sperling paradigm (Block, 2007; Kouider et al., 2010; Lau and Rosenthal, 2011; Vandenbroucke et al., 2014; Cohen et al., 2016; Haun et al., 2017; Bronfman et al., 2019). Some researchers consider that initial subjective impression is a pure illusion; what exists is the only accessed information. Another interpretation is that the initially vague and partial experiences develop into a fuller form when they are cognitively accessed (Kouider et al., 2010).^8^

Various interpretations of the Sperling paradigm are related to a debate in consciousness research on the conceptual distinction of phenomenal consciousness per se (or broad-sense qualia) and cognitively accessible aspects of consciousness (or, behavioral reports) (Block, 2005).

In a sense, the debate around the Sperling paradigm centers around interpretations about (1) the nature of the initial qualia, (2) the reliability and limitation of subsequent behavioral reports about the qualia, and (3) the relationship between the qualia and the reports. As far as we know, these distinctions have been never made explicit with category theoretical concepts, in particular, relationships among qualia or reports as either ~ or ≤. In addition, we believe that a concept of adjunction can clarify the source of the controversy and offer a possible experimental verification. Empirical experiments with the Sperling paradigm demonstrate that reported initial qualia are incomplete and inaccurate with respect to the presented array. Our point is that this fact alone cannot pinpoint if this is caused by the issue in qualia, report, or their relation. To better characterize the situation, our conceptual analysis naturally leads to a proposal to incorporate (A) relational behavioral reports about the initial qualia (such as similarity or inclusion) and (B) qualia reconstruction paradigm, based on a composition of Report;Infer and evaluation of the reconstruction accuracy.

For example, the initial subjective impression of “seeing a whole” is indeed accurately reportable. Furthermore, people can “report” some of the letters accurately. If the latter can be reported with higher confidence, some notion of “order” or “inclusion” may be able to capture the relationships between these reports, which should reflect relations in qualia. From these reports, it is possible to “infer” and reconstruct what they saw in what kind of relationship. Let us say participant A reports “I saw an array of the alphabet with very high confidence. I also saw the letter “Z” with high confidence, but I have very low confidence for other letters.” While this report is still inaccurate and incomplete, it contains reports about graded relationships among the confidence. With such a report, we surmise that the Infer functor conducted by another participant can be meaningfully evaluated by the original participant. The original participant can score how well qualia reconstruction *via* Respond;Infer was successful. Our adjunction hypothesis predicts that the deviation should be “coherent.” Such a novel paradigm may reveal the nature of qualia, report, and their relationship, to advance the field and potentially resolve the controversy.

Different interpretations about the nature of qualia can be modeled using different categorical structures from illusory (e.g., fewer objects, coarser distinctions, and poor relationships) to rich (e.g., much graded objects, finer distinctions, and multitude of relationships). Then, these models can be tested using different categories of behavior reports, ranging from fine verbal reports to coarse button presses with two alternatives.

Critically, we can now explicitly consider the role of functors Report and Infer, as well as their composition, Report;Infer, as a reconstruction paradigm. In the aforementioned original Sperling paradigm, the functor Report has two types; WholeReport and PartialReport. The former just asks the participants what they see after seeing the array of letters. The latter also asks what they see, but gives a specific attentional cue in a part of the display AFTER the array disappears. In this way, we can think of different psychological tasks as different functors. To our knowledge, such a viewpoint has not been proposed in consciousness research [for a related view in psychology and cognitive science, refer to Phillips (2021)].^9^ We believe that this meta-theoretical perspective is useful as a framework, possibly moving the controversy of the rich vs. poor nature of consciousness (Block, 2007; Kouider et al., 2010; Lau and Rosenthal, 2011; Vandenbroucke et al., 2014; Cohen et al., 2016; Haun et al., 2017; Bronfman et al., 2019) to the next stage of empirical research.

As another possible empirical experiment, we can propose “reconstruction” experiments using natural images.^10^ For example, an array of natural images can be briefly presented to the participants asking them what they saw in the image (Chuyin et al., 2022; Qianchen et al., 2022). In other words, this task is considered as a functor Report with conscious experiences as objects in Q and reports as objects in B. Participants can freely report what they saw by typing words (Chuyin et al., 2022). Or they can express whether they saw a patch of an image in the target image or not (Qianchen et al., 2022). To allow for accurate reconstruction of original qualia, reports need to be flexible and rich [such as free reports or drawings (Haun et al., 2017)]. Furthermore, consideration of what to count as an arrow is important. As a start, we can consider that “indistinguishably similar, ~” or some ordered relations “is experienced more or less confidently, ≥” as arrows between objects in both categories. A collection of behavioral reports (objects in B) can now be shown to a separate group of participants, asking them to pick one of the experiences (objects in Q) that match best with the report. How much deviation is there between the original object X and the reconstructed object Report;Infer(X)? While we need to develop some ways to quantify the degree of deviation, in principle, paradigms along this line can establish whether Q (in a broad sense) and B are related in adjunction. Coherency between the two can promote understanding of structures of qualia through behavioral reports, even if they are not identical, isomorphic, or even equivalent.

### 3.3. Significance of adjunction in consciousness research

Finally, we briefly consider a possible application of the concept of adjunction from a viewpoint of consciousness and communication in general.

Traditionally, philosophers have considered the private nature of consciousness as one of the most essential characteristics of consciousness (in particular, its qualitative aspect, or qualia) (Dennett, 1988; Piccinini, 2009). It is true that no one can directly and perfectly share the experience or qualia with anyone else. However, this fact appears inconsistent with another fact that consciousness research has made substantial empirical progress. This is especially enigmatic when considering the “discovery” of a subset of human populations whose phenomenology is substantially different from the rest of the population. A primary example would be the synesthetes (Ramachandran and Hubbard, 2001), whose phenomenological reports used to be untrusted by others in history. However, through accumulated evidence (which we can consider as different functors to different reports), existence and its study are now regarded as legitimate in cognitive neuroscience (Ward, 2013). How can these findings and studies be possible if consciousness is purely private?

A concept of adjunction may provide a formal explanation of this enigma. Even if original qualia are never accurately reported and inferred in different persons, as long as the deviation between the original and the reconstructed is “coherently” related (through a natural transformation), accurate and useful communications can be established. This is formally related to the reconstruction theory in a category of theoretical context (Phillips, 2021).

The idea of reconstruction of other people's qualia through coherently related reports (even if they are incomplete and inaccurate) can be also extended into other domains of communication. For example, children may not be able to accurately describe their thoughts verbally to adults. Compared to adults, their vocabulary may be smaller, with each word meaning different things from the definitions in the dictionary. Still, adults can understand what children are thinking. Our adjunction hypothesis can also be extended in this case. Categories of ChildrenThoughts and AdultThoughts may be related *via* two functors ReportsFromCtoA and ReportsFromAtoC. When two functors are composed, one concept from one category can go to the other category and come back to the original category, with a slight deviation from the original thought. The existence of an adjunction would mean that the deviations are coherent. To obtain coherency, we need a massive number of iterations between people. Similar situations can arise from the category of People in different cultures. In this sense, development and evolution (including cultural evolution) can be regarded as a dynamical and iterative process of public refinement of adjoint relationships between people with private consciousness.

The key idea of our adjunction theory is that the relationship between qualia and reports is “systematically incomplete and inaccurate.” Without such a systematicity, it is hard to see how people could ever have reasonably consistent expectations of the subjective experiences of other people. For example, if we know someone well enough, we can generally predict their likes and dislikes for certain novel situations (e.g., things to eat, places to go). This point is essentially an analog of Fodor's notion of systematicity [also refer to Phillips and Wilson (2010, 2011, 2012)], but here in the context of subjective experience.

In terms of empirical research, it would be ideal to have analysis methods, such as computational algorithms that allow us to find correspondence between structures that are known to be not the same. One possible approach is an optimal transport method (Peyré and Cuturi, 2020). Optimal transport has been used in the field of machine translation without any labels (there is also some connection between optimal transport and enriched category. Refer to https://golem.ph.utexas.edu/category/2021/06/duality_in_transport_problems.html). Recent breakthroughs in this domain demonstrate that a massive number of similarity relationships among words in one language (e.g., English) can find quite accurate correspondence with those in another language (e.g., Japanese) without knowing any labels (Alvarez-Melis and Jaakkola, 2018). Such a method could be introduced in consciousness research, helping find equivalence, and adjoint relationships between structures of qualia and behavioral reports (Kawakita et al., 2023).

A meta-theoretic understanding with adjunction offers a clear picture of the limits and scope of the establishment of consciousness in other minds. In principle, the same idea can be applied to consciousness in babies, nonverbal patients, or even other animals as well. With a concept of adjunction,^11^ hopefully, we would be able to reasonably study and understand the structures of qualia across development and evolution, even if communication is imperfect.

This way of thinking in terms of adjunctions may lead to a new world view. Rather than believing there is one and only globally correct way to view the world, accepting and respecting different perspectives (e.g., categories), which are still locally coherent relationships (e.g., adjoint functors), can enrich the understanding of the world. Indeed, locally consistent but globally inconsistent situations arise in many areas from quantum physics, relational database theory, semantics, and so on (Abramsky, 2020). While discarding a simpler world view may be uncomfortable to some, we believe that facing the complex reality, which is locally consistent but globally inconsistent, with a proper tool would serve for a better understanding of complex human minds. Such an attitude and strategy would lead to the betterment of our quality of life in the future.

## Author contributions

The ideas in the paper originated from the discussions among the authors. NT wrote the first draft and prepared the figures. HS and SP revised the draft. All authors contributed to the article and approved the submitted version.

## Footnotes

^1^Throughout this article, we use the term “adjoint” when we refer to functors (e.g., F is left adjoint to G, F, and G are a pair of adjoint functors, F and G are adjoints, F and G are in an adjoint relationship). When we refer to the entire arrangement, we call it an “adjoint situation” or in short “adjunction”. That is, (F, G, s, t, phi, psi): C⇀D, consisting of: - two functors, F and G (left and right adjoints). - two natural transformations, s and t, (unit and counit of adjunction). - two families of bijections, phi and psi, between hom-sets indexed by the pairs of objects (A, B) in C x D, sometimes called the left and right adjuncts. Usually, an adjunction is given by a subset of that data, which implies the rest, e.g., (F, G, phi): C → D is enough to determine s, t, psi. In that sense, we also write as follows “F and G constitute an adjunction”.

^2^The main difference between adjunction and an isomorphism of categories is that an adjunction identifies a systematic local correspondence between a set of relations among objects rather than a global correspondence between objects which may not exist. In this sense, adjunction can be considered as a weaker type of structural sameness. This precisely weak and local sameness can be found in many examples, such as categories of Sets and Category, Free and Forgetful construction, and the universal morphisms, limits, and colimits (Mac Lane, 1998; Lawvere and Schanuel, 2009; Simmons, 2011; Leinster, 2014).

^3^For simplicity, in the following, we consider achromatic color labels for (B, ≤).

^4^In this mathematical sense, so-called “identity” theories of mind and matter, consciousness and brain do not make sense (Tsuchiya et al., 2016).

^5^Universal morphism is another fundamental concept in category theory (Awodey, 2010) and its relevance to cognitive science has been elaborated in Phillips (2022b).

^6^Other famous definitions of adjunction are based on the definition via hom-sets and the definition via co-unit and unit. While the former clarifies the symmetry in adjunction, it is less fit for the overall theme of this article, where we start off by admitting the asymmetry between qualia and reports. The latter is convenient for mathematicians interested in proof. Mac Lane (1998) defines adjunction using the hom-sets, then gives 5 equivalent definitions (p. 83), including those via universal morphisms and via co-unit and unit. The definition via universal morphism is also introduced as “easy to state, and require minimal verifications when constructing an adjoint functor or proving two functors are adjoint” in Wikipedia (Wikipedia Contributors, 2022).

^7^Why is it possible to match intensity across many types of qualia? This is a curious fact in psychology (Kahneman, 2011), a topic that a category theoretical perspective may be useful to analyze.

^8^While it is not the focus of this article, this standard summary of the Sperling paradigm may not reflect actual phenomenology of participants (e.g., see Cova et al., 2021). In our recent experiments (Chuyin et al., 2022), we showed a natural scene image briefly (followed by a scramble masking image to remove iconic memory) and asked participants to report 5 words that describe what they saw in the image. Among many natural images, we embedded an image of the Sperling array. In this condition, contrary to our expectation, many participants spontaneously reported highly specific individual letters (especially in the top left).

^9^While we will not describe it further in this article, considering each psychological task as a functor is a powerful conceptual tool. In category theory, a functor between two categories can be considered as an arrow in a category, whose objects are categories (so called category of categories, Cat). In this context, a category of Qualia (Q) can be related to various different kinds of categories of Behavioral reports, such as B1, B2, B3, and so on, each of which is related to Q through different functors Report, R1, R2, R3, and so on. While the category of Qualia (Q) itself may not be easy to characterize, its relationships (as functors) with all objects in this category of categories (qualia and behavioral responses, QB) can be characterized. Such an indirect characterization is the same (up to isomorphism) as the direct characterization of Q itself. This is a consequence of the Yoneda lemma, one of the most important concepts in category theory. For details, see Tsuchiya and Saigo (2021) and Tsuchiya et al. (2022).

^10^To be more precise, one needs both the task instances as functors and their relationships as the natural transformations.

^11^Considering various tasks as arrows in category of categories and applying the Yoneda lemma further promotes this idea.
